# Predictive value of serum irisin for chronic heart failure in patients with type 2 diabetes mellitus

**DOI:** 10.1186/s43556-022-00096-x

**Published:** 2022-11-09

**Authors:** Alexander A. Berezin, Ivan M. Fushtey, Sergii V. Pavlov, Alexander E. Berezin

**Affiliations:** 1 Zaporozhye Medical Academy of Postgraduate Education, 20, Vinter av., Zaporozhye, 69096 Ukraine; 2 Department of Therapy and Endocrinology, Zaporozhye Medical Academy of Postgraduate Education, 20, Vinter av., Zaporozhye, 69096 Ukraine; 3 Department Clinical and Laboratory Diagnostics, Zaporozhye State Medical University, 26, Mayakovsky av., Zaporozhye, 69035 Ukraine; 4 Therapeutic Unit, Internal Medicine Department, Zaporozhye State Medical University, 26, Mayakovsky av., Zaporozhye, 69035 Ukraine

**Keywords:** Type 2 diabetes mellitus, Heart failure, Irisin, Natriuretic peptides, Predictive model

## Abstract

We hypothesize that serum irisin can have additional discriminative potency for heart failure (HF) in individuals with type 2 diabetes mellitus (T2DM). The study group comprised 226 consecutive T2DM patients (153 patients with any HF phenotypes and 30 patients without HF) aged 41 to 65 years. The plasma levels N-terminal brain natriuretic pro-peptide (NT-proBNP) and irisin were detected by ELISA at the baseline of the study. We found that the most appropriate cut-off value of irisin (HF versus non-HF) were 10.4 ng/mL (area under curve [AUC] = 0.96, sensitivity = 81.0%, specificity = 88.0%; *P* = 0.0001). Cutoff point of NT-proBNP that distinguished patients with HF and without it was 750 pmol/L (AUC = 0.78; sensitivity = 72.7%, specificity 76.5%, *p* = 0.0001). Using multivariate comparative analysis we established that concentrations of irisin < 10.4 ng/mL (odds ration [OR] = 1.30; *P* = 0.001) and NT-proBNP > 750 pmol/mL (OR = 1.17; *P* = 0.042), left atrial volume index (LAVI) > 34 mL/m^2^ (OR = 1.06; *P* = 0.042) independently predicted HF. Irisin being added to NT-proBNP improved predictive modality for HF, whereas combination of NT-proBNP and LAVI > 34 mL/m^2^ did not. In conclusion, we established that irisin had independent predicted potency for HF in patients with established T2DM.

## Introduction

Type 2 diabetes mellitus (T2DM) remains to be a powerful risk factor for newly heart failure (HF) [1]. Moreover, all-cause mortality, cardiovascular (CV) outcomes, and untoward clinical course of the disease including urgent hospital admission, are considered to have been worse for HF patients with T2DM when compared with those who had no diabetes [2]. Along with it, at least of quarter of symptomatic HF patients with different phenotypes of the condition exerts a persistence of hyperglycemia that contributes to the increased T2DM risk in HF patients [3, 4]. In fact, T2DM frequently coexists with HF and modulates each other [5].

Incidence and prognosis of HF in T2DM patients seem to be potentially predictable factors, which are suggested to be attributes of pathophysiological mechanisms playing pivotal role in various maladaptive responses, such as adverse cardiac remodeling, systemic and microvascular inflammation, endothelial and adipose tissue dysfunctions, oxidative stress, hyperglycemia, lipotoxicity, metabolic memory and altered repair system [6–8]. Being involved in vicious circle of HF pathogenesis, these factors roughly overlap and seem to be accurately evaluated by their surrogate circulating indicators [9]. Although the natriuretic peptides (NP) definitely have powerful diagnostic potency to rule out HF, their predictive ability for HF seem to be sufficient limitation in T2DM patients [10]. It has been previously revealed that a discriminative potency of NP for HF with preserved ejection fraction is appeared to be lower than for HF with reduced ejection fraction [11, 12]. These phenotypes of HF distinguish each other from a signature of comorbidity including metabolic diseases, it is reasonable to suggest that T2DM could be a factor contributing to limiting predictive value of NP for HF in this population. To up to date our knowledge, multiple biological marker models are regarded to be more predictable than single biomarker ones [13].

Irisin is a multifunctional myokine secreted by adipocytes, cardiac myocytes, and skeletal muscle, possibly mediating a wide range of metabolic processes including 'browning' of white adipose tissue, thermogenesis, insulin resistance, muscle endurance, endothelial function, inflammatory and immune reactions and bone osteoblast activity [14, 15]. In physiological condition, circulating irisin positively associates with skeletal muscle mass, glucose homeostasis, skeletal muscle metabolism and negatively with fasting glycaemia [16]. Although muscle and adipose tissue expressions of irisin mRNA were found to be increased in prediabetes, T2DM was associatied with a reduction of the expression and the level of the peptide in plasma [17, 18]. Along with it, HF related to dramatic decrease in circulating irisin levels regardless of a presence of T2DM [19]. There is a hypothesis that irisin acting as peroxisome proliferator-activated receptor gamma coactivator 1-alpha-related protein mediates myocardial contractility and skeletal muscle function and prevent adverse cardiac remodeling, alteration cardiac function and biomechanical stress [20–23]. Thus, dynamic changes of irisin in peripheral circulation seem to be powerful predictor of cardiac remodeling and HF irrespectively from its phenotypes, whereas it remained uncertain whether irisin is comparable to NT-proBNP in its predictive ability in T2DM patients and if add-on of irisin to NT-proBNP is able to improve discriminative potency of the combination. We hypothesize that serum irisin has additional discriminative potency for HF in patients with T2DM.

## Results

### Characteristics of the study patient population

The study patient population composed of mainly male with average age of 51 years and numerous co-morbidities and cardiovascular (CV) risk factors, which included dyslipidemia (83.1%), hypertension (86.3%), stable coronary artery disease (29.5%), smoking (43.2%), and obesity (45.9%) (Table 1). Atrial fibrillation was detected in 17 patients (9.20%) with HF, whereas T2DM patients without HF did not have this condition. LV ventricular hypertrophy, chronic kidney disease and microalbuminuria were found in 73.2%, 21.3% and 30.6%, respectively. Amongs 153 patients with HF 26.2% had HFpEF, 26.8% demonstrated HFmrEF finally HFrEF was detected in 30.6% individuales. All of them had II/III NYHA class. Therefore, mean LVEF was 53.2%, average levels of NT-proBNP were 2718 pmol/mL and irisin were 9.85 ng/mL.

**Table 1:** Characteristics of the study patient population

Variables	Entire T2DM population (*n* = 183)	HF patients (*n* = 153)	Non-HF (*n *= 30)	*P*-value (HF vs non-HF)
Age, year	51 (41–62)	52 (41–64)	51(41–60)	0.86
Male, n (%)	118 (64.5)	100 (65.4)	18 (60.0)	0.82
Dyslipidemia, n (%)	152 (83.1)	127 (83.0)	25 (83.3)	0.84
Hypertension, n (%)	158 (86.3)	132 (86.3)	26 (86.7)	0.80
Stable CAD, n (%)	54 (29.5)	49 (32.0)	5 (16.7)	**0.001**
Atrial fibrillation, n (%)	17 (9.2)	17 (9.2)	0	**0.0001**
Smoking, n (%)	79 (43.2)	63 (41.2)	16 (53.3)	**0.044**
Abdominal obesity,	84 (45.9)	71 (46.4)	13 (43.3)	0.88
Microalbuminuria, n (%)	56 (30.6)	47 (30.7)	9 (30.0)	0.84
LV hypertrophy, n (%)	134 (73.2)	123 (80.3)	11 (36.7)	**0.001**
CKD 1–3 grades, n (%)	39 (21.3)	35 (22.9)	4 (13.3)	**0.001**
HFpEF / HFmrEF / HFrEF, n (%)	48 (26.2) / 49 (26.8) / 56 (30.6)	48 (31.4) / 49 (32.0) / 56 (36.6)	-	-
II/III NYHA class, n (%)	103 (56.3) / 50 (27.3)	103 (67.3) / 50 (23.7)	-	-
BMI, kg/m^2^	25.8 ± 2.1	25.6 ± 2.8	26.3 ± 2.6	0.88
Waist circumference, sm	85.6 ± 2.9	85.1 ± 3.2	86.5 ± 3.1	0.86
WHR, units	0.86 ± 0.03	0.85 ± 0.05	0.87 ± 0.03	0.86
SBP, mm Hg	132 ± 5	129 ± 6	135 ± 5	0.81
DBP, mm Hg	80 ± 4	78 ± 5	84 ± 3	0.80
LVEDV, mL	154 ± 6	161 ± 4	147 ± 6	**0.001**
LVESV, mL	72 ± 7	86 ± 6	59 ± 3	**0.001**
LVEF, %	53 ± 6	46 ± 3	60 ± 2	**0.001**
LVMMI, g/m^2^	151 ± 6.12	154 ± 5	137 ± 3	**0.01**
LAVI, mL/m^2^	39 ± 8	38 ± 4	30 ± 5	**0.042**
E/e`, unit	14.0 ± 0.46	13.5 ± 0.33	7.2 ± 0.42	**0.001**
eGFR, mL/min/1.73 m^2^	83 ± 6.0	75 ± 4.0	86 ± 3.5	**0.01**
HOMA-IR	7.65 ± 3.7	7.95 ± 2.3	7.15 ± 2.4	0.14
Fasting glucose, mmol/L	5.84 (4.61–7.02)	5.62 (4.30–6.96)	5.92 (4.61–6.97)	0.28
HbA1c, %	6.65 ± 0.04	6.59 ± 0.02	6.70 ± 0.05	0.70
Creatinine, mcmol/L	108.8 ± 12.0	108.6 ± 8.5	95.1 ± 10.4	0.26
TC, mmol/L	6.41 (6.15–6.82)	6.43 (6.17–6.70)	6.42 (6.35–6.90)	0.82
HDL-C, mmol/L	0.95 (0.73–1.12)	0.97 (0.80–1.07)	0.93 (0.70–1.10)	0.80
LDL-C, mmol/L	4.43 (4.10–4.75)	4.38 (4.27–4.49)	4.51 (4.35–4.68)	0.68
TG, mmol/L	2.26 (2.11–2.31)	2.21 (2.04–2.39)	2.30 (1.18–3,46)	0.64
NT-proBNP, pmol/mL	2718 (1380 – 3720)	3115 (2380 – 3750)	105 (72 – 142)	**0.001**
Irisin, ng/mL	9.85 (4.34 – 13.60)	6.50 (3.10—10.5)	12.90 (11.2 – 13.4)	**0.001**

*Abbreviations*: *BMI* Body mass index, *CKD* Chronic kidney disease, *DBP* Diastolic blood pressure, *E/e`* Early diastolic blood filling to longitudinal strain ratio, *eGFR* Estimated glomerular filtration rate, *HDL-C* High-density lipoprotein cholesterol, *HFpEF* Heart failure with preserved ejection fraction, *HFmrEF* Heart failure with mildly reduced ejection fraction, *HFrEF* Heart failure with reduced ejection fraction, *LAVI* Left atrial volume index, *LVEDV* Left ventricular end-diastolic volume, *LVESV* Left ventricular end-systolic volume, *LVEF* Left ventricular ejection fraction, *LVMMI* Left ventricle myocardial mass index, *LDL-C* Low-density lipoprotein cholesterol, *SBP* Systolic blood pressure, *TG* Triglycerides, *TC* Total cholesterol, *WHR* Waist-to-hip ratio

We did not notice significant differences between cohorts in demographic and anthropomorphic parameters, presentation of several CV risk factors, and blood pressure. Along with it, HF cohorts composed of more amount of patients with chronic coronary artery disease, LV hypertrophy, chronic kidney disease (1^st^-to-3^rd^ grades) and having smoking habits than non-HF cohorts (*P* = 0.001 for all cases). Therefore, LVEDV, LVESV, LVMMI, LAVI and E/e` were found to be sufficiently higher and LVEF was lower in HF patients than in non-HF individuals. In addition to that, we did not find any difference in HOMA-IR and biochemical parameters between cohorts, apart from NT-proBNP and irisin, the levels of which exerted multidirectional changes. The HF individuals demonstrated significantly higher NT-proBNP and lower irisin than those without HF.

### Spearmen correlation between circulating levels of irisin and other variables

In entire patient cohort irisin levels were positively associated with NYHA class, LVEF, HOMA-IR, NT-proBNP, and HDL-C, whereas negative associations were found for eGFR, WHR, BMI, systolic and diastolic BP, total cholesterol, triglycerides and LDL-C (Table 2). There were no significant correlations of concomitant medication including SGLT2 inhibitors with HOMA-IR and irisin levels. In T2DM patients with HF irisin exhibited moderate correlation with NT-proBNP, LVEF, and BMI, whereas its association with NYHA class, WHR and diastolic BP were weak. Irisin concentrations in non-HF patients correlated positively with HOMA-IR, NT-proBNP, HDL-C and inversely with WHR, BMI, total cholesterol, LDL-C and also eGFR.

**Table 2:** Spearmen correlations between irisin levels and other parameters

Factors that correlate with the levels of irisin	Entire population		HF patients		None-HF patients	
	**r Spearmen**	***P*** **value**	**r Spearmen**	***P*** **value**	**r Spearmen**	***P*** **value**
HOMA-IR	0.34	**0.001**	0.14	0.12	0.44	**0.003**
NT-proBNP	0.33	**0.001**	0.38	**0.001**	0.38	**0.001**
LVEF	0.26	**0.044**	0.32	**0.001**	0.17	0.54
NYHA class	0.24	**0.01**	0.16	0.05	-	-
TC	-0.27	**0.001**	-0.11	0.06	-0.24	**0.042**
HDL-C	0.24	**0.02**	0.13	0.42	0.29	**0.001**
LDL-C	-0.29	**0.001**	-0.13	0.22	-0.31	**0.001**
TG	-0.28	**0.001**	-0.20	0.050	-0.32	**0.001**
eGFR	-0.32	**0.024**	-0.21	0.054	-0.30	**0.001**
WHR	-0.40	**0.001**	-0.25	**0.001**	-0.42	**0.001**
BMI	-0.32	**0.001**	-0.30	**0.001**	-0.34	**0.001**
SBP	-0.28	**0.001**	-0.06	0.26	-0.20	**0.046**
DBP	-0.26	**0.001**	-0.21	**0.001**	-0.22	**0.044**

*Abbreviations*: *BMI* Body mass index, *BP* Blood pressure, *DBP* Diastolic blood pressure, *eGFR* Estimated glomerular filtration rate, *HDL-C* High-density lipoprotein cholesterol, *HOMA-IR* Homeostasis Model Assessment of insulin resistance, *LDL-C* Low-density lipoprotein cholesterol, *LVEF* Left ventricular ejection fraction, *NT-proBNP* N-terminal brain natriuretic pro-peptide, *NYHA* New York Heart Association, *SBP* Systolic blood pressure, *TC* Total cholesterol, *TG* Triglycerides, *WHR* Waist-to-hip ratio

### Predictive pattern of models for all HF phenotypes

**Fig. 1:**
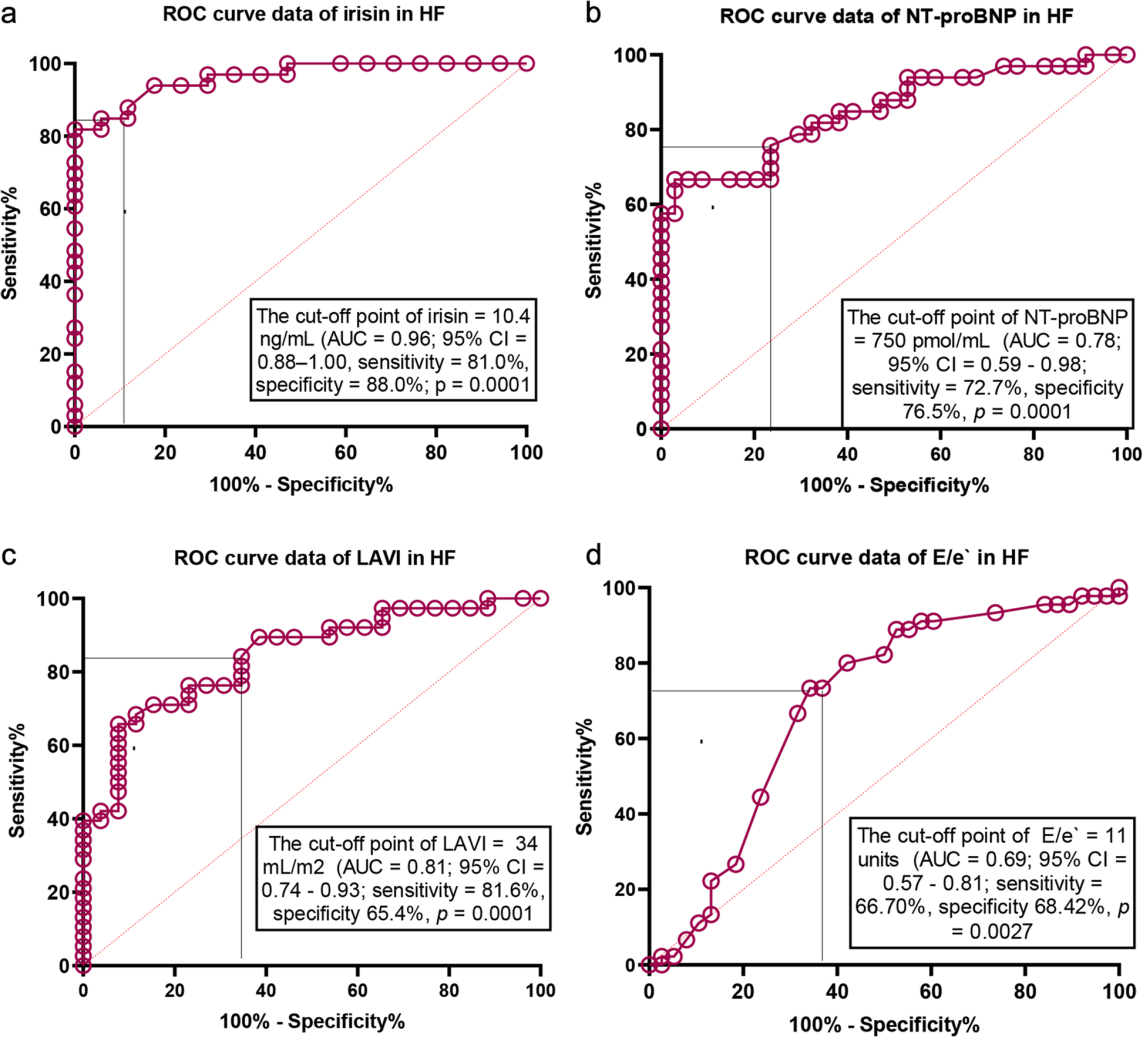
Reliability of the predictive models for HF. The results of ROC curve analysis. **a** ROC curve data of irisin in HF. **b** ROC curve data of NT-proBNP in HF. **c** ROC curve data of LAVI in HF. **d** ROC curve data of E/e` in HF. Abbreviations: AUC, area under curve; CI, confidence interval; HF, heart failure; LAVI, left atrial volume index; E/e`, early diastolic blood filling to longitudinal strain ratio; NT- proBNP, N-terminal brain pro-natriuretic peptide

The reliability of the predictive models for HF is reported Fig. 1. The Receive Operation Characteristics curve analysis yielded that the well-balanced cut-off point for irisin (with HF versus free HF) were 10.4 ng/mL (area under curve [AUC] = 0.96, sensitivity = 81.0%, specificity = 88.0%; *P* = 0.0001) (Fig. 1a). Cutoff point of NT-proBNP that distinguished patients with HF and without it was 750 pmol/L (AUC = 0.78; sensitivity = 72.7%, specificity 76.5%, *p* = 0.0001) (Fig. 1b). Along with it, the best cutoff point for LAVI was 34 ml/m2 (AUC = 0.81; sensitivity = 81.6%, specificity 65.4%, *p* = 0.0001) (Fig. 1c). E/e` (HF versus non-HF) was 11 unit (AUC = 0.69; sensitivity = 66.7%, specificity 68.4%, *p* = 0.0027) (Fig. 1d). Thus, irisin exhibited more profound discriminative potency for HF when compared with NT-proBNP and featured of diastolic LV dysfunction (LAVI, E/e`).

### Logistic regression analysis

Univariate logistic regression model yielded that the predictors for HF in patients enrolled in the study were the following factors: irisin < 10.4 ng/mL, LV hypertrophy, BMI, NT-proBNP > 750 pmol/mL, age, LAVI > 34 mL/m^2^ and E/e’ > 11 (Table 3). Concomitant medications were no remarkable predictors for HF. Multivariate logistic model showed that the serum levels of irisin < 10.4 ng/mL along with NT-proBNP > 750 pmol/mL and LAVI > 34 mL/m^2^ were independent predictors for HF.

**Table 3:** Predictors for HF in T2DM patient populations

	**Logistic Regression Models**			
	**Univariate**		**Multivariate**	
	**OR (95% CI)**	***P*-Value**	**OR (95% CI)**	***P*-Value**
NT-proBNP (> 750 pmol/mL vs ≥ 750 pmol/mL)	1.54 (1.06–2.33)	**0.001**	1.17 (1.02–1.26)	**0.042**
Irisin (< 10.4 ng/mL vs ≥ 10.4 ng/mL)	1.52 (1.16–2.86)	**0.001**	1.28 (1.08–2.15)	**0.001**
LAVI (> 34 mL/m^2^ vs ≤ 34 mL/m^2^)	1.19 (1.11–1.36)	**0.001**	1.06 (1.02–1.13)	**0.044**
LVH (present vs absent)	1.12 (1.06–1.20)	**0.044**	1.05 (1.00–1.11)	0.14
E/e’ (> 11 units vs ≤ 11 units)	1.12 (1.06–1.21)	**0.001**	1.04 (1.00–1.06)	0.42
BMI (present vs absent)	1.07 (1.02–1.11)	**0.046**	1.05 (1.00–1.08)	0.062
Age	1.03 (1.02–1.05)	**0.048**	1.03 (1.00–1.04)	0.16

*Abbreviations*: *BMI* Body mass index, *E/e’* Early diastolic blood filling to longitudinal strain ratio, *LAVI* Left atrial volume index, *LVH* Left ventricular hypertrophy, *NT-proBNP* N-terminal brain natriuretic pro-peptide

### Comparison and reclassification of the predictive values of the models

We established that inclusion of irisin to reference predictive model constructed from NT-proBNP significantly added discriminatory information of the model, whereas LAVI did not exhibit modality to increase the discriminative potency of NT-proBNP (Table 4). Thus, irisin remarkably improved the predictive value of NTproBNP for HF in T2DM patients, whereas we did not find a difference between isolated irisin and irisin + reference model that seems to show an independent discriminative value of irisin for HF.

**Table 4:** The comparisons of predictive models for HF: The results of model fit statistics

Models	Depending variable: HF					
	Area under curve		Net-reclassification improvement		Integrated discrimination indices	
	**M ± SD**	***P*-value**	**M ± SD**	***P*-value**	**M ± SD**	***P*-value**
Reference Model 1 (NT-proBNP)	0.78 ± 0.16	-	-		-	
Model 2 (NT-proBNP + LAVI)	0.82 ± 0.06	0.05	0.15 ± 0.04	0.64	0.17 ± 0.03	0.42
Model 3 (NT-proBNP + irisin)	0.97 ± 0.01	0.0001	0.63 ± 0.02	0.045	0.56 ± 0.03	0.012
Model 4 (Irisin)	0.96 ± 0.01	0.0001	0.53 ± 0.01	0.044	0.42 ± 0.01	0.024

*Abbreviations*: *LAVI* Left atrial volume index, *M* Median, *SD* Standard deviation, *HF* Heart failure, *NT-proBNP* N-terminal brain natriuretic pro-peptide

## Discussion

Our study demonstrated that irisin concentrations were lower in T2DM patients with established HF independently from its phenotypes. Aline with it, well balanced cutoff level for irisin that distinguished HF cohort from non-HF cohort was found to be 10.4 ng/mL. Yet, add-on of irisin level to the predictive HF model constructed from elevated levels of NT-proBNP remarkably exerted its additive potency.

We hypothesize that these findings may be promising for personified transitional management of HF regardless of a signature of comorbidities including T2DM and profile of HF phenotypes. Indeed, previously received data showed that acute / acutely decompensated HF was strongly associated with an increase in both NT-proBNP and irisin, whereas opposite changes of these biomarkers in terms of increase in NT-proBNP and decrease in irisin accompanied chronic HF [24, 25]. Moreover, there is a strong reason to believe that reduced levels of irisin more typical for maladaptive shifts in adverse cardiac remodeling due to inflammation and fibrosis than elevation of NT-proBNP in circulation reflecting non-specific biomechanical stress [26]. Irisin is sometimes referred to as the 'universal indicator' of early stages of cardiac remodeling as it is capable of attenuating hypoxia / ischemia-induced apoptosis of cardiac myocytes and cardiac hypertrophy [27]. Acting via microRNA-19b/AKT/mTOR and AMPK/mTOR signaling pathways, irisin promotes cardiac protective activity and modulate preconditioning [28, 29]. Finally, its ability to suppress oxidative stress and inflammation is considered to be clear explanation of the cause by which circulating levels of irisin was found to be high in acute HF along with natriuretic peptides and other biomarkers of biomechanical stress and myocardial injury [30]. Thus, a trend to decrease in circulating levels of irisin in HF diabetics when compared to non-HF patients with T2DM seems to be a sign of incapability of endogenous repair system to compensate initial myocardial damage, so it illustrates an occurrence of maladaptive stage in cardiac remodeling directly related to T2DM-induced microvascular inflammation, endothelial dysfunction and skeletal muscle myopathy [31, 32].

On the other hand, weak physical endurance in T2DM is regarded to be a plausible cause of low levels of circulating irisin in comparison with healthy individuals [33, 34]. It has been noticed that skeletal muscle expression of irisin in chronic HF inversely correlated to the levels of inflammatory cytokines [34, 35]. Because T2DM is a well-known factor contributing to atherosclerosis, systemic and microvascular inflammation including adipose tissue inflammatory-related remodeling, skeletal muscle dysfunction and persistently exists among HF including HFpEF, irisin can be a promising predictor of cardiac remodeling and HF [36, 37]. This hypothesis has been confirmed in some clinical studies, which yielded optimistic results in respect with the predictive role of irisin for both HFpEF and HFrEF in patients with T2DM or insulin resistance [38, 39]. The findings of our study showed that irisin levels being positively correlated with LVEF did not exhibit strong correlation with several metabolic parameters including fasting glucose, HOMA-IR in HF cohort, whereas in non-HF T2DM patients these associations were noticed. In fact, these data clarify that the initial trigger for irisin production in HF and non-HF populations can be different and that thereby low levels of irisin is considered a surrogate biomarker of advanced cardiac remodeling and the risk of HF manifestation. Indeed, there is strong evidence of the fact that is consisted of this assumption [39, 40].

However, we noticed that irisin being added to NTproBNP was better predictor for HF than LAVI, so metabolic response in T2DM at high risk of HF occurred ahead of hemodynamic left-side heart changes. Aline with it, we established that isolated use of irisin is taken into account to be because the same predictable of HF as its combination with the based model. This clarifies independent discriminative potency of the biomarker and opens new prospective in further discovery of clinical implication of the model. Perhaps, irisin being abundantly produced by skeletal muscles and adipocytes and other tissues out of myocardium may mediate different variants of peripheral tissue dysfunction, which intervene in an exceedingly progression of HF in T2DM [17, 19]. During this context, irisin seems to point out quite optimistic results that deserve investigating in large clinical trials. Indeed, a metabolic signature of HFpEF frequently collides to serious difficulty within the interpretation of its role in prediction of clinical course of the disease [2, 19]. Yet, there is uncertain whether the patients with HFpEF and T2DM could also be beneficially treated and demonstrate a strict resemblance in clinical outcomes having a major difference in irisin. These findings are regarded to be another target for further studies. Taking into consideration the results of several studies of positive impact of irisin on cardiac function and structure [41, 42], we hypothesized that a decrease in irisin concentration is superior to diastolic and systolic cardiac dysfunction markers in prediction of HF. Finally, we received strong evidence of our hypothesis when the full model including NT-proBNP and irisin became more predictable for HF than others.

There are several limitations in the study, the first of them relates to one center open cohorts study design. Yet, we also do not include T2DM patients having history TIA / stroke, also as those with coronary revascularization or other surgical procedures. The second limitation was that we did not use phenotypes of HF (HFpEF, HFrEF and HFmrEF) for further analysis. The last limitation was a lack of serial measures of circulating biomarkers in a protocol of the study. However, we believe that these limitations are not conclusive and allow extrapolating the results of the study on other populations.

## Methods

### Study design

**Fig. 2:**
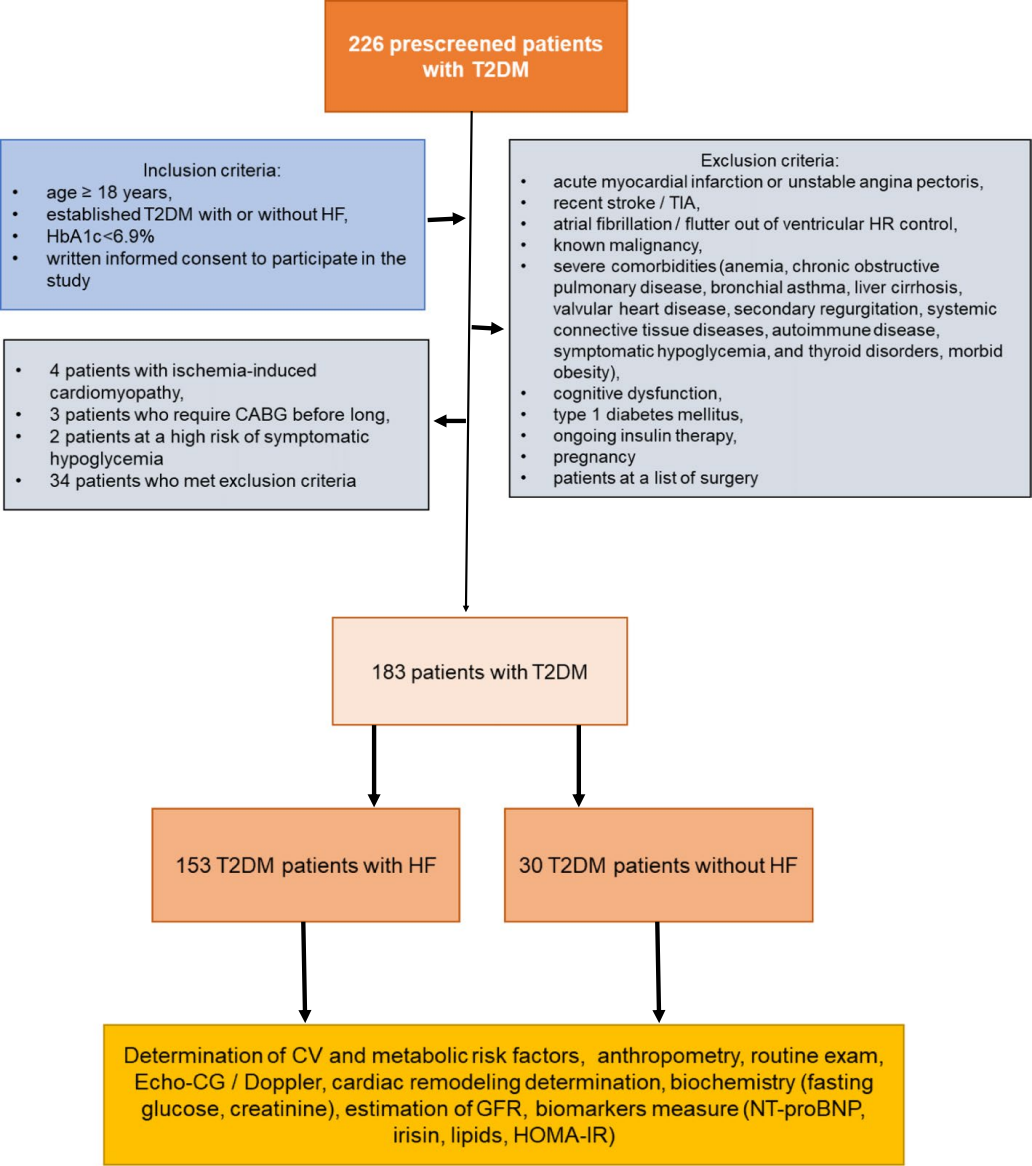
Flow chart of the study design. Abbreviations: CABG, coronary artery bypass grafting; HF, heart failure; HOMA-IR, Homeostatic Assessment Model of Insulin Resistance; HR, heart rate; TIA, transient ischemic attack; T2DM, type 2 diabetes mellitus

A total of 226 T2DM patients who were under investigation in the Private Multidisciplinary Hospital Vita-Centre LTD (Zaporozhye, Ukraine) were consecutively included in the study from October 2020 to December 2021. The following criteria such as age > 18 years, established T2DM regardless of HF, and the levels of HbAc1 less 6.9% were used as inclusion ones. The study design, inclusion / exclusion criteria and reasons to exclusion at the prescreening are reported Fig. 2. Then since the patients were checked in compliance to inclusion / exclusion criteria 183 patients were finally included in the study.

### CV risk factors and co-morbidities

We evaluated CV risk factors and co-morbidities according to current guidelines of the European Society of Cardiology (ESC) [43–46] so as to work out HF during the study we used ESC guidelines [47, 48].

### Routine anthropometric examinations

We evaluated conventional anthropometric parameters including body mass index (BMI), height, weight, and hip-to-waist ratio (WHR) [49].

### B-mode Echocardiography and Doppler examination

Conventional B-mode echocardiography and impulse Doppler examination on Vivid T8 ("GE Medical Systems", Freiburg, Germany) were performed with the aim of elucidating hemodynamic features (left ventricular ejection fraction [LVEF], left atrial volume [LAV], left atrial volume index [LAVI], E/e` ratio) in compliance with current guidelines [50]. Left ventricular hypertrophy (LVH) was detected per conventional echocardiohraphic criteria, including the following: LV mass / body area ≥ 125 g/m2 in male or ≥ 110 g/m2 in female [51].

### Medications

T2DM patients received diet together with personally adjusted dose of metformin and sodium-glucose cotransporter-2 (SGLT2) inhibitor optionally. HF patients were treated in line with conventional clinical recommendation with beta-blockers, antagonists of renin–angiotensin–aldosterone system, loop diuretics, while possible use of SGLT2 inhibitions or i/f blocker ivabradine were also recommended as adjunctive therapies. Lipid-lowering agents were prescribed in subjects with known dyslipidemia, T2DM or established CVD without conventional contraindications. Antiplatelet drugs were added to the therapy when needed.

### Biochemical analyses

Blood samples were collected for biochemical analysis after overnight fasting. After centrifugation (3000 r/min, 30 min) polled serum aliquots were stored at ≤ –70ºC until analysis. Concentrations of irisin and NT-proBNP were measured with commercial ELISA kits (Elabscience, Houston, Texas, USA). All ELISA data were evaluated in step with the quality curve and every sample was measured twice because the norm was finally analyzed. Conventional biochemistry parameters were routinely measured in local laboratory using Roche P800 analyzer (Basel, Switzerland). We used CKD-EPI formula to estimate glomerular filtration rate (GFR) [52]. Insulin resistance was detected as Homeostatic Assessment Model of Insulin Resistance (HOMA-IR) [53].

### Statistical analyses

We used v. 23 SPSS (IBM, Armonk, New York, USA) software and v. 9 GraphPad Prism (GraphPad Software, San Diego, CA, USA) software for statistical analysis. The Kolmogorov–Smirnov test was accustomed evaluate distribution of variables. Baseline characteristics were expressed as counts with percentages or mean ± variance (SD) or median (interquartile range – IQR) for continuous variables and as proportions for categorical variables. For the normally distributed continuous variables, Student’s t-test was used. Otherwise, the Mann–Whitney U-test was chosen instead. We used Spearman coefficient of correlation to represent the connection between variables. With the aim of obtaining a receiver operating characteristic (ROC)-determined biomarker cut-points Youden method was performed. For the optimal cut-of value, the realm under the curve (AUC), 95% confidence Intervals (95% CI), also as a sensitivity and specificity were estimated. Univariate logistic regression was accustomed elucidate plausible predictive factors for HF. The potential candidates (*P* < 0.05) included in multivariate logistic regression. Predictive value of things for HF were confirmed using estimation of integrated discrimination indices (IDI) and net-reclassification improvement (NRI). All the tests performed were 2 sided, and *p* value < 0.05 was considered statistically significant.

## Conclusion

Current predictive model for HF based on measurement of concentrations of natriuretic peptides and detection of cardiac dysfunction or alteration of cardiac structure seems to show the best predictive value for HFrEF, whereas the discriminative potency for HFmrEF/HFpEF is significantly lower than for HFrEF. Therefore, coexisting comorbidities including obesity and T2DM intervene in problematic issue to interpretation of the variability of NT-proBNP. In this connection, discovery of new biomarkers that are able to improve predictive capability of current model is promising. Irisin ensures a metabolic regulation of cardiac function and structure and tackles skeletal muscle activity with common clinical features of HF such as fatigue and low tolerability to physical exercise along with direct impact on adverse cardiac remodeling. Yet, irisin seems to show their predictive potency regardless of NT-proBNP and any HF phenotype.

We established that serum irisin levels were significantly decreased in HF in patients with established T2DM when compared with individuals with T2DM without this condition. Along with it, we established that the concentrations of irisin less than 10.4 ng/mL not only had discriminative information for HF, but also was able to improve the discriminative potency of NTproBNP for the disease in T2DM patients. The irisin seem to show optimistic results in terms of its predictive ability for HF independently from NT-proBNP and cardiac features characterized LV diastolic function (LAVI). This finding seem to have promising importance in discovering new predictive models for HF in these patient populations.

## Data Availability

The data in the present study will be available upon request to the corresponding authors.

## References

[1] Virani SS, Alonso A, Aparicio HJ, Benjamin EJ, Bittencourt MS, Callaway CW (2021). American heart association council on epidemiology and prevention statistics committee and stroke statistics subcommittee. Heart disease and stroke statistics-2021 update: a report from the American heart association. Circulation..

[2] Kenny HC, Abel ED (2019). Heart Failure in Type 2 Diabetes Mellitus. Circ Res.

[3] Yap J, Tay WT, Teng TK, Anand I, Richards AM, Ling LH (2019). ASIAN‐HF (Asian Sudden Cardiac Death in Heart Failure) Registry Investigators, Zile M, McMurray J, Lam CSP. Association of Diabetes Mellitus on Cardiac Remodeling, Quality of Life, and Clinical Outcomes in Heart Failure With Reduced and Preserved Ejection Fraction. J Am Heart Assoc..

[4] Ani C, Shavlik D, Knutsen S, Abudayyeh I, Banta J, O'Brien E (2022). Glycemic status, non-traditional risk and left ventricular structure and function in the Jackson Heart Study. BMC Cardiovasc Disord.

[5] Randhawa VK, Dhanvantari S, Connelly KA (2021). How Diabetes and Heart Failure Modulate Each Other and Condition Management. Can J Cardiol.

[6] Dunlay SM, Givertz MM, Aguilar D, Allen LA, Chan M, Desai AS (2019). American Heart Association Heart Failure and Transplantation Committee of the Council on Clinical Cardiology; Council on Cardiovascular and Stroke Nursing; and the Heart Failure Society of America. Type 2 Diabetes Mellitus and Heart Failure: A Scientific Statement From the American Heart Association and the Heart Failure Society of America: This statement does not represent an update of the 2017 ACC/AHA/HFSA heart failure guideline update. Circulation..

[7] Berezin A (2016). Metabolic memory phenomenon in diabetes mellitus: Achieving and perspectives. Diabetes Metab Syndr.

[8] Schütt K, Müller-Wieland D, Marx N (2019). Diabetes Mellitus and the Heart. Exp Clin Endocrinol Diabetes..

[9] Berezin AE, Berezin AA (2020). Circulating Cardiac Biomarkers in Diabetes Mellitus: A New Dawn for Risk Stratification-A Narrative Review. Diabetes Ther.

[10] Chen L, Huang Z, Zhao X, Liang J, Lu X, He Y (2022). Predictors and Mortality for Worsening Left Ventricular Ejection Fraction in Patients With HFpEF. Front Cardiovasc Med.

[11] Aimo A, Gaggin HK, Barison A, Emdin M, Januzzi JL (2019). Imaging, Biomarker, and Clinical Predictors of Cardiac Remodeling in Heart Failure With Reduced Ejection Fraction. JACC Heart Fail.

[12] Chen H, Chhor M, Rayner BS, McGrath K, McClements L (2021). Evaluation of the diagnostic accuracy of current biomarkers in heart failure with preserved ejection fraction: A systematic review and meta-analysis. Arch Cardiovasc Dis.

[13] Topf A, Mirna M, Ohnewein B, Jirak P, Kopp K, Fejzic D (2020). The diagnostic and therapeutic value of multimarker analysis in heart failure. An approach to biomarker-targeted therapy. Front Cardiovasc Med..

[14] Kurdiova T, Balaz M, Vician M, Maderova D, Vlcek M, Valkovic L (2014). Effects of obesity, diabetes and exercise on Fndc5 gene expression and irisin release in human skeletal muscle and adipose tissue: in vivo and in vitro studies. J Physiol.

[15] Kim H, Wrann CD, Jedrychowski M, Vidoni S, Kitase Y, Nagano K (2018). Irisin Mediates Effects on Bone and Fat via αV Integrin Receptors. Cell.

[16] Waseem R, Shamsi A, Mohammad T, Hassan MI, Kazim SN, Chaudhary AA (2022). FNDC5/Irisin: Physiology and Pathophysiology. Molecules.

[17] Balakrishnan R, Thurmond DC (2022). Mechanisms by Which Skeletal Muscle Myokines Ameliorate Insulin Resistance. Int J Mol Sci.

[18] Akyuz A, Mert B, Ozkaramanli Gur D, Mucip Efe M, Aykac H, Alpsoy S (2021). Association of lower serum irisin levels with diabetes mellitus: Irrespective of coronary collateral circulation, and syntax score. North Clin Istanb.

[19] Berezin AA, Lichtenauer M, Boxhammer E, Fushtey IM, Berezin AE (2022). Serum Levels of Irisin Predict Cumulative Clinical Outcomes in Heart Failure Patients With Type 2 Diabetes Mellitus. Front Physiol.

[20] Korta P, Pocheć E, Mazur-Biały A (2019). Irisin as a multifunctional protein: Implications for health and certain diseases. Medicina.

[21] Schnyder S, Handschin C (2015). Skeletal muscle as an endocrine organ: PGC-1α, myokines and exercise. Bone.

[22] Li RL, Wu SS, Wu Y, Wang XX, Chen HY, Xin JJ (2018). Irisin alleviates pressure overload-induced cardiac hypertrophy by inducing protective autophagy via mTOR-independent activation of the AMPK-ULK1 pathway. J Mol Cell Cardiol.

[23] Boström P, Wu J, Jedrychowski MP, Korde A, Ye L, Lo JC (2012). A PGC1-α-dependent myokine that drives brown-fat-like development of white fat and thermogenesis. Nature.

[24] Ho MY, Wang CY (2021). Role of Irisin in Myocardial Infarction, Heart Failure, and Cardiac Hypertrophy. Cells.

[25] Han X, Zhang S, Chen Z, Adhikari BK, Zhang Y, Zhang J (2020). Cardiac biomarkers of heart failure in chronic kidney disease. Clin Chim Acta.

[26] Prabhu SD, Frangogiannis NG (2016). The biological basis for cardiac repair after myocardial infarction: from inflammation to fibrosis. Circ Res.

[27] Peng Q, Wang X, Wu K, Liu K, Wang S, Chen X (2017). Irisin attenuates H2O2-induced apoptosis in cardiomyocytes via microRNA-19b/AKT/mTOR signaling pathway. Int J Clin Exp Pathol.

[28] Deng J, Zhang N, Chen F, Yang C, Ning H, Xiao C (2020). Irisin ameliorates high glucose-induced cardiomyocytes injury via AMPK/mTOR signal pathway. Cell Biol Int.

[29] Peng Q, Ding R, Wang X, Yang P, Jiang F, Chen X (2021). Effect of Irisin on pressure overload-induced cardiac remodeling. Arch Med Res.

[30] Ou-Yang WL, Guo B, Xu F, Lin X, Li FX, Shan SK (2021). The controversial role of irisin in clinical management of coronary heart disease. Front Endocrinol (Lausanne).

[31] Berezin AE (2019). Endogenous vascular repair system in cardiovascular disease: The role of endothelial progenitor cell. Australasian Medical J.

[32] Paulus WJ, Tschöpe C (2013). A novel paradigm for heart failure with preserved ejection fraction: comorbidities drive myocardial dysfunction and remodeling through coronary microvascular endothelial inflammation. J Am Coll Cardiol.

[33] Armandi A, Rosso C, Nicolosi A, Caviglia GP, Abate ML, Olivero A (2022). Crosstalk between Irisin levels, liver fibrogenesis and liver damage in non-obese, non-diabetic individuals with non-alcoholic fatty liver disease. J Clin Med.

[34] Philippou A, Xanthis D, Chryssanthopοulos C, Maridaki M, Koutsilieris M (2020). Heart Failure-Induced Skeletal Muscle Wasting. Curr Heart Fail Rep.

[35] Matsuo Y, Gleitsmann K, Mangner N, Werner S, Fischer T, Bowen TS (2015). Fibronectin type III domain containing 5 expression in skeletal muscle in chronic heart failure-relevance of inflammatory cytokines. J Cachexia Sarcopenia Muscle.

[36] van Empel V, Brunner-La Rocca HP (2015). Inflammation in HFpEF: Key or circumstantial?. Int J Cardiol.

[37] Guo W, Zhang B, Wang X (2020). Lower irisin levels in coronary artery disease: a meta-analysis. Minerva Endocrinol.

[38] Silvestrini A, Bruno C, Vergani E, Venuti A, Favuzzi AMR, Guidi F (2019). Circulating irisin levels in heart failure with preserved or reduced ejection fraction: a pilot study. PLoS ONE.

[39] Kałużna M, Pawlaczyk K, Schwermer K, Hoppe K, Człapka-Matyasik M, Ibrahim AY (2019). Adropin and irisin: new biomarkers of cardiac status in patients with end-stage renal disease? a preliminary study. Adv Clin Exp Med.

[40] Moreno-Navarrete JM, Ortega F, Serrano M, Guerra E, Pardo G, Tinahones F (2013). Irisin is expressed and produced by human muscle and adipose tissue in association with obesity and insulin resistance. J Clin Endocrinol Metab.

[41] Yan W, Chen Y, Guo Y, Xia Y, Li C, Du Y (2022). Irisin promotes cardiac homing of intravenously delivered MSCs and protects against ischemic heart injury. Adv Sci (Weinh).

[42] Yu Q, Kou W, Xu X, Zhou S, Luan P, Xu X (2019). FNDC5/Irisin inhibits pathological cardiac hypertrophy. Clin Sci (Lond).

[43] Piepoli MF, Hoes AW, Agewall S, Albus C, Brotons C, Catapano AL (2016). 2016 European Guidelines on cardiovascular disease prevention in clinical practice: The Sixth Joint Task Force of the European Society of Cardiology and Other Societies on Cardiovascular Disease Prevention in Clinical Practice (constituted by representatives of 10 societies and by invited experts) Developed with the special contribution of the European Association for Cardiovascular Prevention & Rehabilitation (EACPR). Eur Heart J..

[44] Catapano AL, Graham I, De Backer G, Wiklund O, Chapman MJ, Drexel H (2016). 2016 ESC/EAS Guidelines for the Management of Dyslipidaemias: The Task Force for the Management of Dyslipidaemias of the European Society of Cardiology (ESC) and European Atherosclerosis Society (EAS) Developed with the special contribution of the European Assocciation for Cardiovascular Prevention & Rehabilitation (EACPR). Atherosclerosis.

[45] Williams B, Mancia G, Spiering W, AgabitiRosei E, Azizi M, Burnier M (2018). 2018 ESC/ESH Guidelines for the management of arterial hypertension. Eur Heart J..

[46] Mach F, Baigent C, Catapano AL, Koskinas KC, Casula M, Badimon L (2020). 2019 ESC/EAS Guidelines for the management of dyslipidaemias: lipid modification to reduce cardiovascular risk. Eur Heart J..

[47] Seferovic PM, Ponikowski P, Anker SD, Bauersachs J, Chioncel O, Cleland JGF (2019). Clinical practice update on heart failure 2019: pharmacotherapy, procedures, devices and patient management. An expert consensus meeting report of the Heart Failure Association of the European Society of Cardiology. Eur J Heart Fail..

[48] McDonagh TA, Metra M, Adamo M, Gardner RS, Baumbach A, Böhm M (2021). 2021 ESC Guidelines for the diagnosis and treatment of acute and chronic heart failure. Eur Heart J..

[49] Garvey WT, Mechanick JI, Brett EM, Garber AJ, Hurley DL, Jastreboff AM (2016). Reviewers of the AACE/ACE Obesity Clinical Practice Guidelines. American association of clinical endocrinologists and american college of endocrinology comprehensive clinical practice guidelines for medical care of patients with obesity. Endocr Pract..

[50] Baumgartner H, Hung J, Bermejo J, Chambers JB, Edvardsen T, Goldstein S (2017). Recommendations on the Echocardiographic Assessment of Aortic Valve Stenosis: A Focused Update from the European Association of Cardiovascular Imaging and the American Society of Echocardiography. J Am Soc Echocardiogr.

[51] Nagueh SF, Smiseth OA, Appleton CP, Byrd BF, Dokainish H, Edvardsen T (2016). Recommendations for the Evaluation of Left Ventricular Diastolic Function by Echocardiography: An Update from the American Society of Echocardiography and the European Association of Cardiovascular Imaging. J Am Soc Echocardiogr.

[52] Levey AS, Stevens LA, Schmid CH, Zhang YL, Castro AF, Feldman HI (2009). A new equation to estimate glomerular filtration rate. Ann Intern Med..

[53] Matthews DR, Hosker JP, Rudenski AS, Naylor BA, Treacher DF, Turner RC (1985). Homeostasis model assessment: insulin resistance and beta-cell function from fasting plasma glucose and insulin concentrations in man. Diabetologia.

